# OMENTAL INFARCTION: SURGICAL or CONSERVATIVE TREATMENT? A CASE REPORTS and CASE SERIES SYSTEMATIC REVIEW

**DOI:** 10.1016/j.amsu.2020.06.031

**Published:** 2020-06-27

**Authors:** N.A. Medina-Gallardo, Y. Curbelo-Peña, T. Stickar, J. Gardenyes, S. Fernández-Planas, P. Roura-Poch, H. Vallverdú-Cartie

**Affiliations:** a Department of General Surgery, Hospital Universitari de Vic - Consorci Hospitalari de Vic, Francesc Pla ‘El Vigatà', 1, 08500, Vic, Spain; b Department of Epidemiology, Hospital Universitari de Vic - Consorci Hospitalari de Vic, Francesc Pla ‘El Vigatà', 1, 08500, Vic, Spain

**Keywords:** Omental infarction, Computed tomography, Acute abdominal pain, Conservative treatment, Surgical treatment

## Abstract

**Background:**

Omental infarction (OI) is an infrequent cause of acute abdominal pain and there is no consensus on whether conservative or surgical treatment is the best strategy when performing positive CT diagnosis.

**Objectives:**

To assess which of the two treatments is the most commonly adopted and compare outcomes in terms of success rate in resolution of symptoms and hospital length of stay.

**Eligibility criteria:**

Case report and case series of patients with abdominal pain and positive diagnosis by CT of omental infarction.

**Data sources:**

PubMed, Science Direct and Google Scholar in combination with cross-referencing searches and manual searches of eligible articles from January 2000 to June 2018.

**Participants:**

Patients older than 18 years of age.

**Methods:**

Patient characteristics and results were summarized descriptively. Categorical variables were assessed by chisquare test or Fischer's exact test, and continuous variables by the Wilcoxon-Mann-Whitney or Kruskal-Wallis test. Risk factors for failure of the conservative management were identified using multivariate logistic regression.

**Results:**

90 articles were included in the final analysis (146 patients). 107 patients (73.3%) received conservative treatment with a failure rate of 15.9% (patients needing surgery) and 39 patients (26.7%) received surgery as first treatment. The mean hospital length of stay was 5.1 days for the conservative treatment group and 2.5 days for the surgery group with statistically significant differences (p = 0.00). Younger age and white blood cells count ≥12000/μl were predictive factors of conservative treatment failure.

**Conclusions:**

Although conservative treatment is effective in most patients, surgery has advantages in terms of hospital length of stay.

## Highlights

• Conservative treatment seems to be the preferred option when making a CT diagnosis of omental infarction.
• However, it fails in 15% of patients, needing surgery sometimes with worse local conditions due to evolutive complications.
• Surgical treatment, especially through the laparoscopic approach, appears to be safe and involves a shorter hospital stay.

## 1 Introduction

Omental infarction (OI) is a rare cause of acute abdominal pain. Since the first case was described by Eitel in 1899, more than 300 cases have been published [1,2].

The clinical diagnosis remains challenging without complementary tests, due to its clinical similarity with other more frequent causes of acute abdominal pain. Most of the time the OI involves the right side of the omentum, therefore 90% of the cases [3] are diagnosed intraoperatively in acute abdomen, when assessing patients for more common pathologies such as acute appendicitis or cholecystitis.

There are two main pathological mechanisms that can lead to OI: secondary to the vascular pedicle torsion on its own axis, or due to situations that predispose to thrombosis as hypercoagulable states or vascular abnormalities.

Consequently, both situations lead to a vascular compromise of the area of the omentum affected, producing haemorrhagic extravasation, with bloody fluid, necrosis and adhesions [4].

OI as a result of vascular pedicle torsion, can be divided into primary or secondary: the first without underlying pathology; whereas the second (responsible for approximately two thirds of the cases) [4], due to the presence of an intra-abdominal pathologic process that makes the point of distal “anchorage” of the omentum (cysts, tumours, intra-abdominal inflammatory foci, previous surgical wounds or hernia sacs) [5].

Cases reported range from the paediatric age [[6], [7], [8]] to elderly patients5, although most cases appear in people between 30 and 50 years old, with predominance in male and obese patients [9].

The usual symptom is continuous, localized abdominal pain, with increasing intensity, while nausea and vomiting are variable [6]. About half of patients present with low-grade fever and middle leucocytosis in blood tests. While most have a single episode of abdominal pain, some patients may suffer recurrent pain, which may be related with intermittent twisting of the omentum. Initial clinical diagnosis usually assesses to appendicitis, cholecystitis, diverticulitis or complicated ovarian cyst [10], and mesenteric adenitis or complicated Meckel's diverticulum in paediatric patients. However, patients with OI appear to be less affected and having less signs of inflammatory response than other acute abdominal processes [4].

The increasing use of CT has made preoperative diagnosis more common. Hence management becomes a challenge.

Accumulated experience is mainly based on isolated clinical cases where both, conservative and surgical management, have been advocated as the best option of treatment. Therefore, when diagnosis of OI is made, the most appropriate treatment remains controversial.

We carried out a systematic review of published cases of OI diagnosed by CT (excluding those with intra-abdominal pathology associated) where the main goal was to assess the most commonly adopted treatment and its results.

## 2 Material and Methods

This review was undertaken and reported in accordance with the PRISMA (Preferred Reporting Items for Systematic Reviews and Meta-Analyses) and AMSTAR (Assessing the methodological quality of systematic reviews) Guidelines [11,27].

### 2.1 Eligibility criteria

We reviewed case reports and series of cases with a diagnosis of OI. To be included, the published cases had to meet the following inclusion criteria: (i) patients 18-years-old with abdominal pain and positive CT diagnosis of OI, (ii) absence of associated abdominal pathology, and (iii) describing the treatment chosen, and its results. Cases in which the radiological description was consistent with OI but no explicit mention of the diagnosis was made, were not considered. Radiologically diagnosed but asymptomatic patients were also excluded.

### 2.2 Search strategies and information sources

All available studies about OI were reviewed from January 2000 to June 2018. A comprehensive search comprising keywords and MeSH was carried out in PubMed. In addition, a manual search was also made in Science Direct and Google scholar with “Omental infarction” [TW] (words in the title) for the same time period (Table 1). A subsequent search was performed from cited articles in the initial search. There were no restrictions in languages, and assessment of quality studies was not performed.

**Table 1:** Search strategy.

DATABASE	Search strategy
PubMED	1. Omentum [MeSH] 2. Infarction [MeSH] 3. Torsion [TW] 4. Infarction [TW] 5. “Omental infarction” [TIAB] 6. “Omental torsion” [TIAB] 7. (Omentum [MeSH] AND Infarction [MeSH]) NOT Surgery [MeSH] 8. Adult [MeSH] OR Aged [MeSH] OR “Aged, 18 and over” [MeSH] a) 1 AND 2 AND (3 OR 4) AND 8 b) 1 AND 2 AND (5 OR 6) AND 8 c) 7 AND 8
ScienceDirect	“Omental infarction” [TW]
Google Scholar	“Omental infarction” [TW]

### 2.3 Study selection

Search strategies were implemented by AM. After eliminating duplicates, the remaining articles and abstracts were evaluated for inclusion. The relevant articles were recovered and independently evaluated by two groups of authors (YC & JG, and TS & SF). Disagreements between authors were resolved by another author (HV) and if necessary final adjudication was made by the senior author (AM).

### 2.4 Data collection process and data items

Using Microsoft Excel Version 2016 (Microsoft Corporation, Redmond, WA), relevant data was extracted independently by the two author groups (as above) and compared. Discrepancies were discussed with AM and HV as adjudicators. PR was in charge of checking data, processing and analysing results. Data from articles published in languages other than English, French, Portuguese, German or Spanish, were extracted if abstract was available in one of the aforementioned languages. Extracted data included year of publication, demographic characteristics, clinical presentation, treatment chosen (conservative or surgical), and results for each patient described. For pooled data in case series articles, the summary statistics and the percentages presented were collected and were attributed to each of the individuals in the series.

As primary outcome we considered conservative treatment compared with surgical treatment in terms of success of resolution of symptoms and hospital length of stay. As secondary outcome we considered duration of symptoms, fever, leucocytosis and surgical approach (including rate of conversion from laparoscopy to laparotomy) in the cases of surgical treatment. Additionally, patients from the conservative management group were compared according to success or failure of this strategy.

In order to perform statistical analysis, outcomes provided descriptively were considered in numerical values according to the current practice definitions in our centre (based on Haematology and Hemotherapy Spanish Society and American Association for Clinical Chemistry) as follows: for white blood cell count, “normal” was considered as < 12000/μl, whereas “leucocytosis” or “moderate leucocytosis” was considered as ≥ 12000/μl. For temperature, “afebrile”, “low grade fever” or “febricula” were considered <37.5 °C, although “fever” or “febrile” were considered as ≥ 37.5 °C. Number of patients from whom data were obtained are indicated in brackets in the section results. All patients were analysed on an intention-to-treat basis.

### 2.5 Summary measures and statistical analysis

Statistical analysis was performed using SPSS v.23 (IBM Corp., Armonk, NY). Patient characteristics, disease manifestations and results were summarized descriptively. Categorical variables were assessed by chi-square test or Fischer's exact test, and continuous variables by the Wilcoxon-Mann-Whitney or Kruskal-Wallis test. Risk factors for failure of the conservative management were identified using multivariate logistic regression. A p value ≤ 0.05 was considered statistically significant.

## 3 Results

**Fig. 1:**
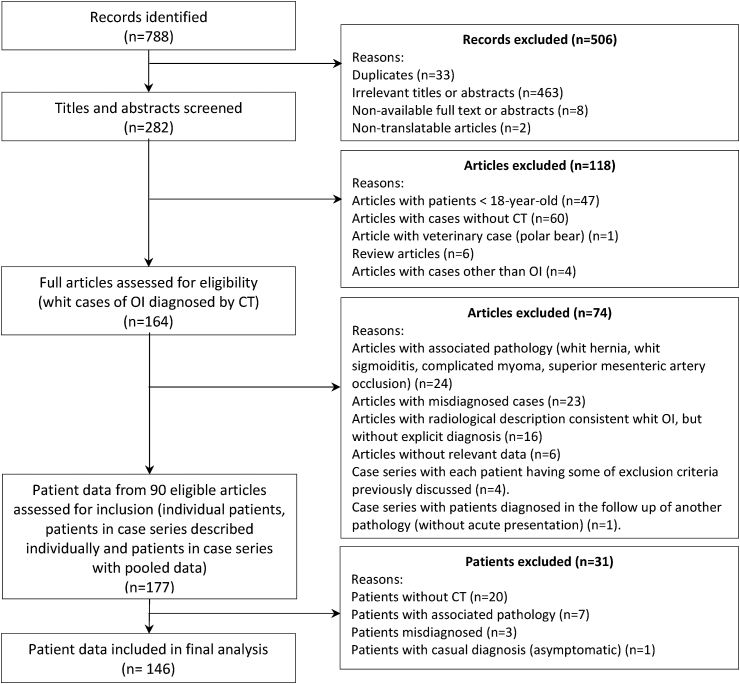
PRISMA diagram describing the article and patient selection process.

After removing non-relevant articles, 282 articles with OI were retained, of which 90 were assessed. Among these articles, after eliminating the cases that did not meet inclusion criteria, 146 patients were included for the final analysis. The PRISMA diagram describing the selection process is presented in Fig. 1 and cases are summarized in Table 2. The list of articles included is presented in the supplementary material (Appendix A).

**Table 2:** Cases and extracted data.

	Year	Author	Gender	Age (years)	Duration of symptoms	Temperature (°C)	Blood white cell count (μL-1)	Treatment	Conservative treatment failure	Surgical approach	Hospital stay (days)
1	2018	Alzahrani et al.	F	50	24h	36,4	12300	Conservative	No	–	n/a
2	2018	Coulier	M	76	n/a	n/a	n/a	Conservative	No	–	n/a
3	2018	Alshehri et al.	M	46	n/a	n/a	n/a	Conservative	No	–	n/a
4	2018	Udechukwu et al.	M	61	92h	*afebrile*	*n/a*	Conservative	No	–	n/a
5	2018	Ong et al.	M	27	*acute*	*fever*	17900	Conservative	No	–	7
6	2018	Criado-Martin et al.	M	86	24h	37,2	8920	Conservative	No	–	6
7	2017	Choh	M(2 pt) F(3 pt)	42 (28–50)	n/a	n/a	n/a	Conserv. (5 pt)	1 of 5 patients	n/a	n/a
8	2017	Buell et al.	F	58	48h	*afebrile*	*normal*	Surgery	–	Laparoscopy	2
9	2017	Snachez-López-Gay et al.	M	72	2h	n/a	*leucocytosis*	Conservative	No	–	10
10	2017	Mayoral-López et al.	F	25	72h	*afebrile*	n/a	Conservative	No	–	n/a
11	2017	Suresh et al.	M	24	96h	n/a	12500	Conservative	No	–	5
12	2016	Kolandaivelu et al.	F	55	72h	n/a	n/a	Surgery	–	Laparotomy	n/a
13	2016	Rangarajan et al.	n/a (4 pt)	n/a	n/a	n/a	n/a	Surgery (4 pt)	–	Laparoscopy	n/a
14	2016	Bagul et al.	M	42	7d	n/a	*leucocytosis*	Surgery	–	Laparotomy	n/a
15	2016	Mendoza-Moreno et al.	F	60	48h	38,2	*leucocytosis*	Surgery	–	Laparotomy	2
16	2016	Yu et al.	F	43	*several days*	36,9	9390	Surgery	–	Laparoscopy	2
17	2016	Dutkiewicz et al.	M	37	72h	36,6	*normal*	Conservative	No	–	1
18	2016	Cremonini C et al.	M	28	96h	*afebrile*	*normal*	Surgery	–	Laparoscopy	2
19	2015	Amo-Alonso et al.	F	65	n/a	38	*leucocytosis*	Conservative	No	–	7
20	2015	Ravindradas et al.	M	53	72h	n/a	*12600*	Conservative	No	–	2
21	2015	Aiyappan et al.	M	30	*acute*	*afebrile*	*normal*	Conservative	No	–	n/a
22	2015	Shinde et al.	M	45	48h	37,5	10600	Surgery	–	Laparoscopy	2
23	2015	Litzau et al.	M	38	48h	n/a	*normal*	Conservative	No	–	1
24	2015	Chauhan et al.	F	68	7d	*afebrile*	12800	Conservative	Yes (after 2 weeks)	Percutaneous drainage	11
25	2015	Abbas et al.	F	38	48h	*afebrile*	n/a	Conservative	No	–	n/a
26	2015	Agarwal et al.	M	40	2–3months	*afebrile*	n/a	Conservative	Yes (after 4 weeks; absces formation).	Laparoscopy (conversion to laparotomy)	n/a
27	2015	Sanchez-Fuentes et al.	M	59	n/a	n/a	n/a	Conservative	No	–	5
			F	50	n/a	n/a	n/a	Conservative	No	–	9
			F	49	n/a	n/a	n/a	Conservative	No	–	11
28	2014	El Sheikh et al.	n/a (5 pt)	n/a	n/a	n/a	n/a	Surgery (5 pt)	–	Laparoscopy	n/a
29	2014	Nataraj-Naidu et al.	M	nr	48h	*afebrile*	*normal*	Surgery	–	Laparoscopy	1
30	2014	Occhionorelli S et al.	M	29	72h	37,2	10300	Conservative	Yes (worsening pain after 12 h)	Laparoscopy (conversion to laparotomy)	5
31	2014	Zaafouri et al.	M	20	n/a	37	11500	Conservative	Yes (fever and worsening pain)	Laparoscopy	n/a
32	2013	Wang et al.	M	49	*acute*	*afebrile*	n/a	Conservative	No	–	n/a
33	2013	Katagiri et al.	M	18	n/a	37,4	10100	Conservative	Yes (worsening pain after 48 h)	Laparoscopy (conversion to laparotomy)	9
34	2013	Ryan et al.	M	58	n/a	n/a	n/a	Surgery	–	Laparotomy	n/a
35	2013	George et al.	M	27	*acute*	*febrile*	*leucocytosis*	Conservative	No	–	5
36	2013	Schmidt et al.	M	61	72h	n/a	11400	Conservative	No	–	n/a
37	2013	Le Roux et al.	F	55	n/a	n/a	11000	Surgery	–	Laparoscopy	n/a
38	2012	Bouilland et al.	M	28	24h	*afebrile*	*normal*	Conservative	No	–	1
39	2012	Sable et al.	F	50	48h	n/a	n/a	Surgery	–	Laparotomy	5
40	2012	Park et al.	M	56	72h	*afebrile*	6960	Conservative	No	–	5
			M	57	48h	*afebrile*	12650	Conservative	Yes (no improvement after 7 days)	Laparoscopy	14
			M	52	2h	*afebrile*	10330	Conservative	No	–	3
			M	52	24h	*afebrile*	6550	Conservative	No	–	7
41	2012	Hosseinpour et al.	M	30	n/a	36,8	9000	Conservative	Yes (fever and worsening pain after 12 h)	Laparotomy	5
42	2012	Khouli et al.	F	67	72h	*febricula*	*normal*	Conservative	No	–	n/a
43	2012	Ishimaru et al.	F	75	48h	37,5	11400	Conservative	No	–	6
44	2012	Kerr et al.	M	57	n/a	n/a	n/a	Conservative	No	–	n/a
			F	74	n/a	n/a	n/a	Conservative	No	–	n/a
			M	74	n/a	n/a	n/a	Conservative	No	–	n/a
			F	58	n/a	n/a	n/a	Conservative	No	–	n/a
45	2012	Araújo-Filho et al.	F	36	5d	*afebrile*	12000	Conservative	No	–	n/a
46	2011	Park et al.	F	65	n/a	*afebrile*	17090	Conservative	No	–	5
47	2011	Bersou et al.	M	25	n/a	*afebrile*	*normal*	Conservative	No	–	n/a
48	2011	Kim et al.	F	30	72h	n/a	*normal*	Conservative	No	–	n/a
49	2011	Lopez-Rubio et al.	M	29	n/a	n/a	*normal*	Surgery	–	Laparoscopy	3
50	2011	Barai et al.	M	32	24h	n/a	*normal*	Conservative	No	–	8
51	2011	Modaghegh et al.	F	74	96h	n/a	9500	Conservative	No	–	9
52	2011	Hsu et al.	M	24	72h	n/a	*normal*	Conservative	Yes (no improvement after 3 days)	Laparoscopy	6
53	2011	Rebai et al.	F	65	48h	38,2	14000	Conservative	Yes. (no improvement)	Laparoscopy	n/a
54	2011	Benaghmouch et al.	M	31	92h	38	12300	Surgery	–	Laparoscopy	1
55	2010	Doganay et al.	M	33	2h	n/a	12730	Surgery	–	Laparotomy	n/a
56	2010	Soobrah et al.	F	20	7d	*afebrile*	13600	Conservative	No	–	3
57	2010	Wong et al.	M	53	24h	*afebrile*	12500	Surgery	–	Laparoscopy	n/a
58	2010	Le Moigne et al.	F	52	48h	*afebrile*	11500	Conservative	No	–	5
59	2010	Itenberg et al.	M	32	6h	*afebrile*	14290	Conservative	Yes (after 24 h)	Laparoscopy	1
60	2010	Portillo et al.	M	63	72h	*afebrile*	9000	Surgery	–	Laparoscopy	2
61	2010	Tandon et al.	M	41	96h	37,9	13500	Conservative	No	–	n/a
62	2010	Fernández-Rey.	M	43	48h	*afebrile*	*normal*	Conservative	No	–	n/a
63	2009	Yoon et al.	F	51	72h	36,6	7950	Conservative	No	–	7
64	2009	Maternini et al.	F	40	n/a	n/a	*leucocytosis*	Conservative	Yes.	Laparoscopy	3
65	2009	Bestman et al.	M	41	n/a	*afebrile*	7400	Surgery	–	Laparoscopy	2
66	2009	Franklin Jr et al.	M	63	72h	*afebrile*	9000	Surgery	–	Laparoscopy	2
67	2008	Bessoud et al.	F	70	3weeks	*afebrile*	n/a	Conservative	No	–	n/a
68	2008	Cianci et al.	M	33	24h	*afebrile*	12050	Conservative	Yes (after 3 days)	Laparoscopy	9
			F	52	n/a	n/a	7490	Surgery	–	Laparoscopy	6
69	2008	Auguste et al.	F	56	*acute*	n/a	n/a	Conservative	No	–	n/a
70	2007	Ergun et al.	M	35	72h	37,6	*leucocytosis*	Surgery	–	n/a	n/a
71	2007	Rao et al.	M	29	48h	*afebrile*	5810	Conservative	No	–	n/a
72	2007 2007	Sammour et al.	M	25	n/a	39	n/a	Surgery	–	Laparotomy	5
			M	20	24h	*afebrile*	n/a	Surgery	–	Laparotomy	n/a
			M	32	18h	37,5	n/a	Conservative	No	–	3
			M	25	9d	37	n/a	Conservative	Yes (no improvement after 24 h).	n/a	n/a
			M	26	92h	37,5	n/a	Conservative	No	–	5
73	2007	Lapsia et al.	M	38	*acute*	*afebrile*	n/a	Conservative	No	–	n/a
74	2007	Papaziogas et al.	M	36	72h	37,5	12200	Conservative	Yes (no improvement after 8 h)	n/a	6
75	2006	Coulier	F	72	n/a	n/a	10000	Conservative	No	–	n/a
76	2006	Goh et al.	M	39	48h	38,2	12900	Conservative	No	–	3
77	2006	Coppin et al.	M	36	24h	37,5	21000	Surgery	–	Laparoscopy	2
78	2005	El Hajj et al.	F	38	72h	n/a	*normal*	Surgery	–	Laparotomy	n/a
79	2005	Bachar et al.	F	31	5d	37	5800	Conservative	No	–	n/a
			M	75	48h	36,3	7450	Conservative	No	–	n/a
			F	79	48h	37	10000	Conservative	No	–	n/a
			F	27	48h	37,2	13000	Conservative	No	–	n/a
			M	48	72h	36,3	13900	Conservative	Yes (no improvement pain)	Laparoscopy	n/a
			F	31	24h	37,1	12000	Conservative	No	–	n/a
80	2005	Kerem et al.	M	36	*acute*	37,8	7800	Surgery	–	Laparoscopy	1
81	2004	Atar et al.	F	64	*several hours*	n/a	12000	Surgery	–	Laparotomy	n/a
82	2004	Coulier et al.	F	51	n/a	n/a	5900	Conservative	No	–	n/a
83	2003	Naffaa et al.	F	37	48h	*afebrile*	*normal*	Surgery	–	Laparotomy	n/a
84	2003	Paroz et al.	n/a (3 pt)	n/a	n/a	n/a	n/a	Conserv. (3 pt)	No	–	n/a
			n/a (1 pt)	n/a	n/a	n/a	n/a	Conserv. (1 pt)	Yes (no improvement pain)	Laparoscopy	n/a
85	2003	Saju et al.	M	30	7d	*fever*	10500	Conservative	No	–	n/a
86	2002	Miguel-Perelló et al.	M	38	36h	*afebrile*	*normal*	Conservative	No	–	4
			M	38	48h	*afebrile*	*normal*	Conservative	No	–	4
			M	34	72h	*afebrile*	*normal*	Conservative	No	–	4
			F	50	12h	*afebrile*	*normal*	Conservative	No	–	7
			F	25	72h	*afebrile*	*normal*	Conservative	No	–	3
			M	23	18h	*afebrile*	*normal*	Conservative	No	–	2
87	2002	Coulier et al.	M	55	5d	n/a	n/a	Surgery	–	n/a	n/a
			M	66	*acute*	n/a	n/a	Conservative	No	–	n/a
			M	58	*acute*	n/a	n/a	Conservative	No	–	n/a
			F	80	*acute*	n/a	n/a	Conservative	No	–	n/a
			F	40	*acute*	n/a	n/a	Conservative	No	–	n/a
			F	41	*acute*	n/a	n/a	Surgery	–	n/a	n/a
88	2001	Schwartzman et al.	M	58	*acute*	n/a	n/a	Conservative	No	–	n/a
89	2001	Miguel et al.	n/a (11 pt)	n/a	n/a	n/a	n/a	Conserv. (11 pt)	No	–	n/a
			F (1 pt)	46	n/a	n/a	n/a	Surgery (1 pt)	–	n/a	n/a
90	2001	McClure et al.	n/a (4 pt)	n/a	n/a	n/a	n/a	Conserv. (4 pt)	No	–	n/a

**Fig. 2:**
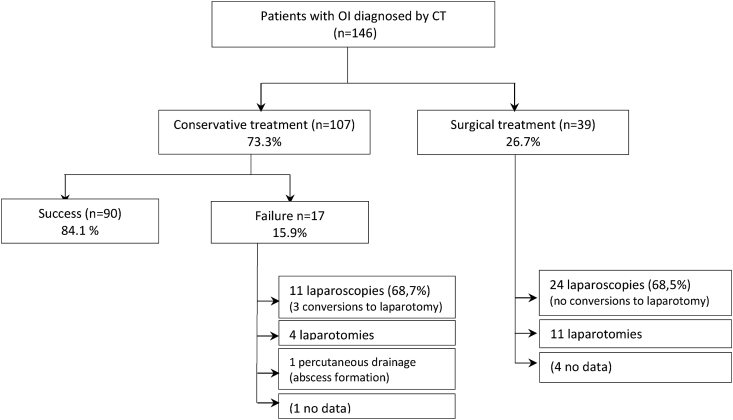
Flowchart of patients with CT diagnosis of OI.

The mean age (data from 117 patients) was 45.7 years old (DS±16.2). 38,9% patients were women and 61,1% were men (data from 113 patients). 107 patients (73.3%) received conservative treatment and 39 (26.7%) surgery as first treatment. Failure rate for conservative treatment was 15.9% (17 patients: 15 for unsolved pain and 2 for abscess formation in the follow up). No postoperative complications were reported in the surgery group, nor mortality in both groups. The flowchart of patients is presented in Fig. 2. The mean age for conservative treatment group (data from 88 patients) was 46.1 years (DS±17.3) and 44.6 years (DS±12.5) for the surgical treatment group (data from 29 patients) with no significant differences. There were no differences in terms of gender.

On admission, 80.0% of patients in the conservative treatment group (data from 65 patients) and 78.3% in the surgical treatment group (data from 23 patients) had less than 72 h of abdominal pain, without significant differences.

Patients with ≥37.5 °C were 7.1% in the conservative treatment group (data from 56 patients) and 29.4% in the surgical treatment group (data from 17 patients) with statistically significant differences (p < 0.05). White blood cell count in the conservative group was ≥12000/μl in 33.9% (data from 59 patients), and in 31.8% for the surgical treatment group (data from 22 patients), without significant statistical differences.

Concerning hospital length of stay, the average was 5.52 days for the conservative treatment group (data from 42 patients) and 2.50 days for the surgical treatment group (data from 16 patients), with statistically significant differences (p = 0.00). Basal characteristics of groups and results are summarized in Table 3.

**Table 3:** Basal characteristics and results.

	Conservative treatment group (n = 107)	Surgical treatment group (n = 39)	*p*
Age	46.1 years (DS 17.3)	44.6 years (DS 12.5)	NS
Gender			
*Male*	73.9%	26.1%	NS
*Female*	72.7%	27.3%	
Duration of abdominal pain			
*< 72 h*	80,0%	78.3%	NS
*> 72 h*	20.2%	21.7%	
Temperature			
*< 37.5°C*	92.9%	70.6%	*0.027*
*≥ 37.5°C*	7.1%	29.4%	
Leucocytosis			
*< 12000*	66.1%	68.2%	NS
*> 12000*	33.9%	31.8%	
Hospital stay	5.52 days	2.50 days	*0.00*

Hospital length of stay was longer when conservative treatment failed, but without significant difference compared to patients in whom conservative treatment was successful (6.9 vs 5.1 days).

In the multivariate analysis, we only detected a younger age (37.9 years, DS± 15.1 vs 47.9 years DS±17.3, p = 0.035) and a higher frequency of white blood cell count ≥ 12000 (61.5% vs 26.1%, p = 0.02) when conservative treatment failed. No difference in terms of evolution time of pain or temperature on admission was found. Comparison between patients with successful or failure on initial conservative treatment is presented in Table 4.

**Table 4:** Comparison between patients with successful and failure on initial conservative treatment.

	Conservative treatment success (n = 90)	Conservative treatment failure (n = 17)	*p*
Age	47.9 years (DS 17.3)	37.9 years (DS 15.1)	*0.035*
Gender			
*Male*	76,5%	23.5%	NS
*Female*	90.6%	9.4%	
Duration of abdominal pain			
*< 72 h*	81.5%	72.7%	NS
*> 72 h*	18.5%	27.3%	
Temperature			
*< 37.5°C*	93%	92.3%	NS
*≥ 37.5°C*	7%	7.7%	
Leucocytosis			
*< 12000*	73,9%	38.5%	*0.02*
*> 12000*	26,1%	61.5%	
Hospital stay	5.1 days	6.9 days	NS

Among surgical treatment group 68.5% patients underwent a laparoscopic approach (data from 35 patients), no cases of conversion to laparotomy were reported. Patients undergoing surgery after failure of conservative treatment, the rate of laparoscopic approach was similar (68.7%) but with a conversion rate to laparotomy of 27.2% (data from 16 patients) (Fig. 2).

## 4 Discussion

Since the first patient described by Eitel in 1899 [26], several of the articles reviewed consider that 250–400 cases of OI have been published [1,[4], [5], [6],^9^,^21^]. However, only in the period of our review, we detected about 250 articles on the subject including more than 300 cases of OI. That means that maybe OI is more common than previously thought, even if it continues to be a rare cause of acute abdominal pain.

With the increasing use of CT, OI has become more frequently diagnosed as the sole cause of acute abdominal pain since its radiological characteristics are well recognized.

However, many cases are diagnosed during exploratory laparotomies or laparoscopies because other common causes of acute abdominal pain such as cholecystitis or appendicitis are suspected in the first place. Additionally, OI can be associated to other abdominal conditions, as an example, most of the times a complicated groin hernia, that requires urgent surgery, carries strangulated content.

As a non-infectious inflammatory condition, the best treatment for patients without an associated intra-abdominal pathology becomes a challenge, since surgery or conservative treatment are the two possible strategies.

The aim of this review is to assess which of the two treatments is the most frequently used when OI is diagnosed by CT, and its results in terms of resolution of symptoms and hospital length of stay.

Soobrah et al. [22] presents a review of literature including 64 patients (pediatric and adults) managed conservatively with a failure rate of 15.6%, and subsequently treated with laparoscopic resection. In a case series article, Kerr et al. [23] describes symptomatic and asymptomatic cases of OI diagnosed by CT following colonic resection, where all patients with abdominal pain were treated successfully with conservative measures. Bachar et al. [24] also describes 6 cases, where only one patient needed surgery due to persistent abdominal pain. Additionally, Miguel-Perelló et al. [25] presents a series of 6 patients diagnosed by CT, all of them treated conservatively.

To the best of our knowledge, our review is the longest recorded, based on published cases on adults. Conservative treatment was the treatment of choice in the most of cases (73.3% of patients), with a high rate of success in resolution of symptoms (84.1%). However, when surgical treatment is chosen, hospital length of stay is shorter (2.5 days vs 5.5 days, p = 0.00), being the longest when conservative treatment fails (5.5 vs 6.9 days, p = NS). In addition, patients in whom conservative treatment failed and underwent laparoscopic surgery, were more likely to need conversion to laparotomy (27.2%), this was not observed in the surgical treatment group. Concerning predictive factors for conservative treatment failure, younger patients (37.9 years, DS± 15.1 vs 47.9 years DS±17.3, p = 0.035) and/or a white blood cell count ≥ 12000 at admission, seem to be related to a higher probability of need for surgery. Although temperature ≥37.5 °C was not observed as a predictor of failure, this is partly explained to the fact that fever at admission makes patients more likely to receive surgical treatment since the beginning.

Authors who advocate for surgical treatment argue that surgery leads to a faster resolution of symptoms and faster recovery, without need of follow-up. These points seem to be clear in our review where in one hand, patients undergoing surgery are discharged earlier, and on the other hand, some patients from the conservative treatment group needed up to 3 months of clinical and radiologic follow-up [[12], [13], [14], [15], [16], [17], [18]]. In addition, surgical treatment can prevent future complications such as abscess formation or intra-abdominal adhesions. However, we were able to detect only two cases of such complications in our review. According to Agarwal et al. [19] one patient underwent surgical intervention because of abscess formation in the follow-up one month after conservative treatment. Likewise, Chauhan et al. [20] describes the same complication after 2 weeks of follow-up, resolving with a percutaneous drainage.

This review presents the typical limitations of an analysis based on isolated clinical cases or small series of cases: lack of prospective design, randomization and masking. We decided to include only patients with a positive diagnosis of OI by CT to assess which is the most commonly adopted treatment. Nevertheless, we were unable to rule out the possibility of missing some important cases pooled in larger series, given that some data was unavailable. Regarding rest of the outcomes (duration of pain, temperature, leucocytosis, hospital stay) not all articles provide the analysed data. Several outcomes can be considered an stimation from all patients diagnosed with OI in both conservative and surgery group. In addition, we have not performed a cost-effectiveness analysis between treatments, so we are not in a position to affirm that although surgical treatment implies a shorter hospital stay, it compensates for the cost of surgery.

In conclusion, findings from the current review help to ascertain that surgical treatment of OI is better than the conservative treatment in terms of hospital length of stay and quicker resolution of symptoms, avoiding complications and need of follow-up. When it comes to comorbidities, patient preferences and laparoscopic experience of the surgical team should also be considered for the decision-making process. Regarding conservative treatment failure, surgeons must be prepared for resection of the omentum, preferably by laparoscopic approach.

## Ethical approval

Ethical approval was considered unnecessary for this study.

## Sources of funding

This was an investigator-initiated study supported by internal funding.

## Author contribution

- **Study design:** Nolberto Adrián MEDINA-GALLARDO. MD, PhD
- **Articles evaluation and data collection:** Yuhamy CURBELO-PEÑA MD, Tomas STICKAR MD, Julia GARDENYES-MARTINEZ MD, Sara FERNANDEZ-PLANAS MD.
- **Disagreement Resolution**: Nolberto Adrián MEDINA-GALLARDO. MD, PhD, Helena VALLVERDU-CARTIE. MD, PhD
- **Data analysis:** Pere ROURA-POCH MD
- **Writing:** Nolberto Adrián MEDINA-GALLARDO. MD, PhD, Helena VALLVERDU-CARTIE. MD, PhD

## Registration of research studies

1. Name of the registry: OMENTAL INFARCTION: SURGICAL OR CONSERVATIVE TREATMENT? A CASE REPORTS AND CASE SERIES SYSTEMATIC REVIEW
2. Unique Identifying number or registration ID: **reviewregistry750**
3. Hyperlink to the registration (must be publicly accessible): https://www.researchregistry.com/browse-the-registry#registryofsystematicreviewsmeta-analyses/registryofsystematicreviewsmeta-analysesdetails/5dab3a4b8da22400157eda2f/

## Guarantor

Nolberto Adrián MEDINA-GALLARDO. MD, PhD.

Helena VALLVERDU-CARTIE. MD, PhD.

Pere ROURA-POCH MD.

## Provenance and peer review

Not commissioned, externally peer reviewed.

## Declaration of competing interest

The researchers involved in this study have no conflicts of interest to declare.
