# Supplementary material for: OMENTAL INFARCTION: SURGICAL or CONSERVATIVE TREATMENT? A CASE REPORTS and CASE SERIES SYSTEMATIC REVIEW
Source: Ann Med Surg (Lond). 2020 Jun 27;56:186–93. doi: 10.1016/j.amsu.2020.06.031 (PMC7334794; doi:10.1016/j.amsu.2020.06.031)
Supplement: Multimedia component 1 [file mmc1.pdf]

APPENDIX A. LIST OF ARTICLES INCLUDED.

1. Abbas M, Stokes L. Omental Infarction masquerading as anastomotic stricture. Surg Obes Relat Dis 2015;11:109-110.
2. Agarwal S, Shaikh A, Navare MS, Singh A, Tayade MB. Primary omental infarction presenting as a parietal wall swelling: a rare case report. J Med Sci Clin Res. 2015;03(08):7267-7270.
3. Aiyappan SK, Ranga U, Veeraiyan S. Omental infarct mimicking acute pancreatitis. Indian J Surg. 2015;77(3):1393–1394.
4. Alshehri M, Khalifa H, Alqahtani A, Aburahmah M. Secondary Omental Infarction in a Patient with a Hypercoagulable. BMJ Case Rep [Internet].2018;2018:bcr-2017-223212.
5. Alzahrani MS, Samman MA, Turkistani ZA. Omental infarction: a rare cause of an acute abdomen in the emergency department: a case report. Int J. Adv. Res. 2018;6(1):445-448.
6. Amo Alonso R, de la Peña Cadenato J, Loza Vargas A, Santos Santamarta F, Sánchez-Ocaña Hernández R, Arenal Vera JJ. Infarction of the greater omentum. Case report. Rev Esp Enferm Dig. 2015;107(11):706-707
7. Araújo Filho JAB, Martines JAS, Martines BMR, Lourenção RM, Pinto LEA, Castro CC. Segmental omental infarction: an unusual cause of acute abdomen. Autopsy Case Rep. 2012;2(2):43–47.
8. Atar E, Herskovitz P, Powsner E, Katz M. Primary Greater Omental Torsion: CT Diagnosis in an Elderly Woman. IMAJ. 2004;6(January):57–8.
9. Auguste T, Roux Y Le, Brefort J, Piquet M, Samama G. Infarctus épiploïque après by-pass gastrique. J Chir. 2008;4:390–1.
10. Bachar GN, Shafir G, Postnikov V, Belenky A, Benjaminov O. Sonographic Diagnosis of Right Segmental Omental Infarction. J Clin Ultrasound. 2005;33(2):76–9.
11. Bagul M, Mehra R, Kumar S, Sharma SPrimary Greater Omental Torsion: Pre-operative CT and Intraoperative Findings Correlation. JCR. 2016;6(3):306-309
12. Barai KP, Knight BC. Diagnosis and management of idiopathic omental infarction: A case report. Int J Surg Case Rep. 2011;2(6):138–140.
13. Benaghmouch F, Aalala EM, Hrora A, Benamer A, Sabbah F, Ahallat M, et al. Acute abdomen for omental torsion. Eur J Radiol. 2011;79:55–57.
14. Bersou M, Heylen Ch, Matthys P, Pringot J. Focal infarction of lesser omentum. JBR–BTR. 2011;94:356.
15. Bessoud B, Buffet C. Infarctus omental segmentaire. Presse Med. 2008;37:1178–1179.
16. Bestman TJR, Valk J W , Gypen B , DeClercq S, Hendrickx L. An Unusual Complication after Roux-en-Y Gastric Bypass: Torsion and Infarction of the Divided Omentum. Obes Surg. 2009; 19:1731–1733.

17. Bouilland O, Le Goudevezem S, Barbe O, Moncade F, Ann X. Infarctus du grand épiploon. Presse Med. 2012;41(9):881-882.

18. Buell KG, Burke-smith A, Patel V, Watfah J. Omental Infarction : The Great Impersonator. Cureus. 2017;9(12):e1940.

19. Chauhan V, Stephenson JA, Shah V. Intra-abdominal focal fat infarction of the omentum: diagnosis and percutaneous management. Br J Radiol Case Reports. 2015;1:20150134.

20. Choh NA, Shera TA, Jabeen S, Ashraf O, Khan AM. Primary and secondary omental infarction : A 5 - year experience in a tertiary care hospital. Saudi Surg J. 2017;5(2):77–81.

21. Cianci R, Filippone A, Basilico R, Storto ML. Idiopathic segmental infarction of the greater omentum diagnosed by unenhanced multidetector-row CT and treated successfully by laparoscopy. Emerg Radiol. 2008;15:51–6.

22. Coppin T, Lipsky D. Twisting and Infarction of the Entire Greater Omentum Managed by Laparoscopy : a Report of Two Cases. Acta chir belg. 2006;106(2):215–7.

23. Coulier B, Pringot J. Infarction of the greater omentum: Can us and CT findings help to avoid surgery? JBR-BTR. 2002;85:193–9.

24. Coulier B, Hoof M Van. Intraperitoneal fat focal infarction of the lesser omentum : case report. Abdom Imaging. 2004;29(March):498–501.

25. Coulier B. A new case of intraperitoneal fat focal infarction (IFFI) of the lesser omentum. Eur Radiol. 2006;16:954–5.

26. Coulier B. Spontaneous and rapid healing of massive symptomatic postoperative right-sided infarction of the greater omentum. Diagn Interv Imaging. 2018;99(5):339-340.

27. Cremonini C, Bertolucci A, Tartaglia D, Menonna F, Galatioto C, Chiarugi M. Acute abdomen caused by greater omentum torsion : A case report and review of the literature. Ulus Travma Acil Cerrahi Derg. 2016;22(4):391–394

28. Criado Martín I, Andrino Díaz NF, López-Dóriga Bonnardeaux P. Infarto de omento: una causa inusual de abdomen agudo. Rev Esp Geriatr Gerontol. 2018;53(2):117–118.

29. Doganay S, Gul Y, Kocakok E. Omental Torsion and Infarction Depicted by Ultrasound and Computed Tomography : An Unusual Cause of. Intern Med. 2010; 49(9):871-872.

30. Dutkiewicz P, Kraleti S, Jarrett D. Omental infarction presenting as abdominal pain typical for cholecystitis. Onc Gas Hep Resp. 2016; 5(1):40–42.

31. El Hajj II, Otrock ZK, Sharara AI. Primary Omental Torsion: Radiologic Diagnosis in a young woman. Dig Dis Sci. 2005;50(6):1169.

32. El Sheikh H, Abdulaziz N. Primary torsion of the greater omentum: Color Doppler sonography and CT correlated with surgery and pathology findings. Egypt J Radiol Nucl Med; 2014;45:19-24.

33. Ergun T, Lakadamyali H, Karabulut Z, Dogan T. Omental torsion without a whirl sign. Australas Radiol. 2007;51:159–60.

34. Fernández-Rey CL. Infarto omental primario como causa de abdomen agudo no quirúrgico : diagnóstico por imagen. Rev Esp Enferm Dig. 2010;102(8):498–499.

35. Franklin ME, Salgado Cruz L, Portillo Ramila G, Díaz Elizondo JA. Tratamiento laparoscópico de la torsión primaria de epiplón. Avances. 2009;6(19):4–7.

36. George R, Leopold K, Prasad K, Nair V. Omental infarction: A misleading cause of an acute abdomen. Saudi Surg J. 2013;1(2):46-8.

37. Goh BK, Heng-Nung K. Non-operative management of idiopathic segmental infarction of the greater omentum successfully diagnosed by computed tomography. J Gastroenterol Hepatol. 2006;21:1634–9.

38. Hosseinpour M, Abdollahi A, Jazayeri H, Talari HR, Sadeghpour A. Omental torsion after repeated abdominal blunt trauma. Arch Trauma Res. 2012;1(2):75-78.

39. Hsu BC, Chou D. Primary idiopathic segmental infarction of the greater omentum. Formos J Surg. 2011;44(6):233–236.

40. Ishimaru N, Maeno T. Omental infarction triggered by tight pants. Intern Med. 2012;51:2235-2237.

41. Itenberg E, Mariadason J, Khersonsky J, Wallack M. Modern Management of Omental Torsion and Omental Infarction : A Surgeon ʼs Perspective. JSurg. 2010;67(1):44–47.

42. Katagiri H, Honjo K, Nasu M, Fujisawa M, Kojima K. Omental infarction due to omental torsion. Case Rep Surg. 2013;1-3.

43. Kerem M, Bedirli A, Mentes BB, Sakrak O, Pala I, Oguz M. Torsion of the Greater Omentum : Preoperative Computed Tomographic Diagnosis and Therapeutic Laparoscopy. JSLS. 2005;9:494–6.

44. Kerr SF, Hyland R, Rowbotham E, Chalmers AG. Postoperative omental infarction following colonic resection. Clin Radiol; 2012;67:134-139.

45. Khouli M, Ribas Batllori MA, Crespo Spósito PE. Causa infrecuente de abdomen agudo. Form Médica Contin en Atención Primaria. Elsevier; 2012;19(10):637.

46. Kim HC, Yang DM, Jin W, Joh JH. Infarction of Lesser Omental Fat Mimicking an Exophytic Pancreatic Tumor by Sonography. J Clin Ultrasound. 2011:412–414.

47. Kolandaivelu PG, Lakshmana R, Balamurugan R, Arun S. Primary omental torsion - A rare case report. IAIM. 2016;3(3):169–171.

48. Lapsia S, Ghai S. Omental infarction: a rare cause of acute abdominal pain. Emerg Med J. 2007;24:779.

49. Le Moigne F, Lamboley J, Vitry T, Bourilhon N, Salamand P, Farthouat P, et al. Une cause rare d ʼ abdomen aigu. Rev Med Interne. 2010;31(10):716–717.

50. Le Roux F, Gennuso F, Lipsker A, Mauvais F. Omental torsion, a rare cause of acute surgical abdomen. J Visc Surg. Elsevier Masson SAS; 2013;150:421-422.

51. Litzau M, Lall MD. Idiopathic left upper quadrant omental infarction: diagnosed and managed conservatively in the ED. Am J Emerg Med. 2015;33:741.e1-741.e2.

52. Lopez Rubio MA, Martinez Ruiz Y.Una causa infrecuente de Dolor Abdominal: El Infarto de Omento. Rev Clin Med Fam. 2011;4(3):254–255.

53. Maternini N, Pezzetta E, Martinet O. Laparoscopic approach for idiopathic segmental infarction of the greater omentum. Minerva Chir. 2009;64:225-227.

54. Mayoral C, Gallego Á, Domínguez M. ¿ Dolor abdominal …? ¿… qué tal un infarto omental ? Form médica Contin en atención primaria. 2017;24(10):576–80.

55. Mendoza Moreno F, Díez Gago MR, Córdova García DM, Pedraza Muñoz A, Díez Alonso M, Noguerales Fraguas F, et al. Primary omental torsion as presentation of acute abdomen. Case Report. Rev Esp Enferm Dig. 2016;108(2):105-106.

56. Mcclure MJ, Khalili K, Serrazin J, Hanbidge A. Radiological Features of Epiploic Appendagitis and Segmental Omental Infarction. Clin Radiol. 2001;56:819–27.

57. Miguel A, Ripollés T, Martinez J, Virgilio M, Ruiz A. Apendicitis epiploica e infarto omental . Hallazgos en ecografía y tomografía computarizada. Radiologia. 2001;43(8):395–401.

58. Miguel Perelló J, Aguayo Albasini JL, Soria Aledo V, Aguilar Jiménez J, Flores Pastor B, Candel Arenas M, et al. Torsión de epiplón : las técnicas de imagen pueden evitar intervenciones innecesarias. Gastroenterol Hepatol. 2002;25(8):493–6.

59. Modaghegh MS, Jafarzadeh R. Primary Omental Torsion in an OldWoman: Imaging Techniques Can Prevent Unnecessary Surgical Interventions. Case Rep Med. 2011;2011:1-3.

60. Naffaa LN, Shabb NS, Haddad MC. CT findings of omental torsion and infarction Case report and review of the literature. J Clin Imaging. 2003;27:116–8.

61. Nataraj Naidu R, Ramalingeswar K, Hemanth V, Satish Kumar R, Madhushankar L. Omental infarction: an unusual cause for acute abdomen. Int J Healthc Sci. 2014;2(1):116-8.

62. Occhionorelli S, Zese M, Cappellari L, Stano R, Vasquez G. Acute abdomen due to primary omental torsion and infarction. Case Rep Surg. 2014;1-4.

63. Ong WM, Matheson J, Chandra R, Stella DL. Omental infarction: a case of a whole omental infarct. ANZ Journal of Surgery. 2018;88(1-2):107–108.

64. Papaziogas B, Dragoumis D, Tsiaousis P, Giakoustidis D, Atmatzidis S, Sarlis G, et al. Primary torsion of the greater omentum. An obscure and unusual cause of acute abdomen. Chirurgia (Bucur). 2018;102(1):95–8.

65. Park TU, Oh JH, Chang IT, Lee SJ, Kim SE, Kim CW, et al. Omental infarction: case series and review of the literature. J Emerg Med. 2012;42(2):149-154.

66. Park KE, Chung DJ, Kim W, Hahn S, Lee JM. Secondary Omental Infarction Related to Open and Laparoscopic-Assisted Distal Gastrectomy : Report of Two Cases. Korean J Radiol 2011;12(6):757–760.

67. Paroz A, Halkic N, Pezetta E, Martinet O. Idiopathic Segmental Infarction of the Greater Omentum : A Rare Cause of Acute Abdomen. J Gastrointest Surg. 2003;7(6):805–8.

68. Portillo GR, Salgado Cruz LES, Franklin ME. Laparoscopic treatment of primary omental torsion : Case report and literature review. Asian J Endosc Surg. 2010;3:39–41.

69. Rangarajan M, Palanivelu C. A Rare Cause of Acute Abdomen Due to Primary Omental Torsion : Value of Laparoscopy in Diagnosis and Treatment. Hellenic J Surg. 2016;88(2):102–105.

70. Rao A, Remer EM, Phelan M, Hatem SF. Segmental omental infarction. Emerg Radiol. 2007;14:195–7.

71. Ravindradas S, Ogunbayo G, Kulandhaisamy S. Omental Infarction : A Rare Cause of Acute Abdominal Pain. Imaging. 2015;148(4):4–5.

72. Rebai W, Ksantini R, Chebbi F, Makni A, Chenitir A, Ayadi S, et al. Infarctus idiopathique du grand épiploon. Tunis Med. 2011;89(2):214-215.

73. Ryan J, Simpson P, McLaughlin S. Gastrointestinal: Omental infarction. J Gastroenterol Hepatol. 2013;28:378.

74. Sable S, Gandhi V, Nagral S, Nagral A. Secondary omental torsion. Br Med J Case Reports. 2012;1-3.

75. Saju X, Priya J. Torsion of the greater omentum with inguinal hernia. Indian J Gastroenterol. 2003;22(September-October):194–6.

76. Sammour KY, Abburu RS, Sammour T. Omental torsion in adults : A clinical twister. Surg Pract. 2007;11:66–70.

77. Sánchez Fuentes PA, López López V, Febrero B, Ramírez P, Parrilla Paricio P. Infarto omental: ¿manejo quirúrgico o conservador? Cir. Esp. 2015;93(7):475-477.

78. Sánchez-López-Gay J, Becerra-Almazán JM, Reyes-Aguilar R, Rodríguez-Barón B, Navarro-Duarte JC. Causa de abdomen agudo no quirúrgico: infarto omental. SEMERGEN - Medicina de Familia. 2017;43(7):534–536.

79. Schmidt S, Juchems M, Kramer K. Eine seltene Differenzialdiagnose des akuten Abdomens. Der Chir. 2013;84(9):780-4.

80. Schwartzman GJ, Jacobs JE, Birnbaum BA. Omental infarction as a delayed complication of abdominal surgery. J Clin Imaging. 2001;25:341–3.

81. Shinde J, Pandit S, Fernandes A, Joshi V, Naik R. Case report of primary omental torsion. Med J DY Patil Univ. 2015;8(4):534–536.

82. Soobrah R, Badran M, Smith SG. Conservative Management of Segmental Infarction of the Greater Omentum : A Case Report and Review of Literature. Case Rep Med. 2010;2010:1-4.

83. Suresh CC, Manappallil RG, Chand A, Jayaraj J. Omental infarction: An uncommon aetiology for acute abdomen. Asian Journal of Medical Sciences. 2017;8(1):104.

84. Tandon AA, Lim KS. Torsion of the greater omentum : A rare preoperative diagnosis. Indian J Radiol Imaging. 2010;20(4):294–297.

85. Udechukwu NS, Souza RSD, Abdulkareem A. Computed tomography diagnosis of omental infarction presenting as an acute abdomen. Radiol Case Reports. 2018;13(3):583–585.

86. Wang W, Wang ZJ, Webb EM, Westphalen AC, Gross AJ, Yeh BM. Omental infarction preceded by anatomically upturned omentum. J Clin Imaging 2013;37:1125-7.

87. Wong EMF, Ka SYJ, Chau WK, Tsui P. Left-sided Torsion of the Greater Omentum. J Hong Kong Col Radiol. 2010;13:154-157.

88. Yoon SJ, Park YD, Chung YJ, Lee SY, Kim ES, Park SY, et al. A case of omental infarction successfully managed with conservative treatment. Korean J Med . 2009;77:36–40.

89. Yu JS, Lee WS, Kim YH. Primary Torsion of Lesser Omentum Presented with Acute Abdomen and Successfully Managed with Laparoscopic Surgery. Chin Med J (Engl). 2016;129(13):1625-1626.

90. Zaafouri H, Elfeki F, Mrad S, Maamer A Ben, Bouhafa A, Cherif A. Idiopathic segmental infarction of the greater omentum. J Surg Pakistan. 2014;19(4):162-3.
